# Metric Formulæ

**Published:** 1891-11

**Authors:** 


### Therapeutic Notes.

---

### METRIC FORMULÆ.

Adopted from the prescriptions of various authorities to this system by A. L. Benedict, M. D., Buffalo, N. Y.

| | |
|---|---|
| ℞.—Iodi | 1.00 |
| Potassii iodidi | 10.00 |
| Glycerini | 25.00 |
| Aquam ad | 100.00 |

In spray or on applicator to nose, pharynx, etc.

---

| | |
|---|---|
| ℞.—Plumbi acetatis | 1.00 |
| Tr. opii | 10.00 |
| Aquam ad | 100.00 |

Lead and opium wash for bruises, cellulitis, etc.

---

| | |
|---|---|
| ℞.—Sodii bicarb | 2.00 |
| Sodii (quadra) boratis | 2.00 |
| Potassii chloratis | 5.00 |
| Ac. carbolici | .50 |
| Glycerini | 25.00 |
| Aquam ad | 100.00 |

Astringent, Dobell's solution.

| | |
|---|---|
| ℞.—Felis bovis inspiss. | 10.00 |
| Ext. conii | 3.00 |
| Saponis duri | 5.00 |
| Olei olivæ q. s. ad | 50.00 |

To absorb hypertrophies.

---

| | |
|---|---|
| ℞.—Tr. belladonnæ | 10.00 |
| Lin. chloroformi | 30.00 |
| Lin. ammoniæ | 30.00 |
| Lin. saponis ad | 100.00 |

Liniment to combine counter-irritation with relief of pain.

---

| | |
|---|---|
| ℞.—Ichthyol | 30.00 |
| Ung. hydrargyri | 30.00 |
| Ung. belladonnæ ad | 100.00 |

First stage of tuberculosis of joints, epididimitis, etc.

---

| | |
|---|---|
| ℞.—Ac. salicylici | 3.00 |
| Ac. boracici | 20.00 |
| Aquam ad | 1000.00 |

Thiersch solution, antiseptic for peritoneal cavity, eye, etc., where there is danger of absorption, or of irritant local action of mercury.

---

| | |
|---|---|
| ℞.—Ung. hydrargyri nitratis | 2.50 |
| Petrolati | 25.00 |

Itching piles, locally.

---

| | |
|---|---|
| ℞.—Ung. gallæ | |
| Ung. hydrarg. ammon. āā | 10.00 |

Bleeding piles, locally.

---

| | |
|---|---|
| ℞.—Potassii chloratis | 1.00 |
| Tr. opii | 1,50 |
| Aq. amyli | 15.00 |

Injection, to be retained for local action on hemorrhoids.

---

| | |
|---|---|
| ℞.—Ac. borici | 1.50 |
| Glycerini | 15.00 |
| Aquam ad | 100.00 |

Use as spray in catarrhal pneumonia, dry bronchitis, etc., to render secretion less viscid.

